# Long-Term Effects of a Comprehensive Intervention Strategy for Salt Reduction in China: Scale-Up of a Cluster Randomized Controlled Trial

**DOI:** 10.3390/nu16234092

**Published:** 2024-11-27

**Authors:** Min Liu, Jianwei Xu, Yuan Li, Feng J He, Puhong Zhang, Jing Song, Yifu Gao, Shichun Yan, Wei Yan, Donghui Jin, Xiaoyu Chang, Zhihua Xu, Yamin Bai, Ning Ji, Ningning Pan, Jing Wu

**Affiliations:** 1 National Center for Chronic and Non-Communicable Disease Control and Prevention, Chinese Center for Disease Control and Prevention (China CDC), Xicheng District, Beijing 100050, China; liumin@ncncd.chinacdc.cn (M.L.); xuajianwei@ncncd.chinacdc.cn (J.X.); baiyamin@ncncd.chinacdc.cn (Y.B.); jining@ncncd.chinacdc.cn (N.J.); ningpan@163.com (N.P.); 2 The George Institute for Global Health, Peking University Health Science Centre, Haidian District, Beijing 100600, China; yli@georgeinstitute.org.cn (Y.L.); zpuhong@georgeinstitute.org.cn (P.Z.); 3 Wolfson Institute of Population Health, Barts and The London School of Medicine & Dentistry, Queen Mary University of London, London E1 4NS, UK; f.he@qmul.ac.uk (F.J.H.); jing.song@qmul.ac.uk (J.S.); 4 Department of Chronic Disease Control and Prevention, Hebei Province Center for Disease Control and Prevention, Shijiazhuang 050024, China; ggyf2008@126.com; 5 Department of Chronic Disease Control and Prevention, Heilongjiang Province Center for Disease Control and Prevention, Harbin 150030, China; yan208@163.com; 6 Department of Chronic Disease Control and Prevention, Jiangxi Province Center for Disease Control and Prevention, Nanchang 330029, China; ggyyderen@126.com; 7 Department of Chronic Disease Control and Prevention, Hunan Province Center for Disease Control and Prevention, Changsha 410028, China; mbk@nhcdc.com; 8 Department of Chronic Disease Control and Prevention, Sichuan Province Center for Disease Control and Prevention, Chengdu 610044, China; changxyu2009@163.com; 9 Department of Chronic Disease Control and Prevention, Qinghai Province Center for Disease Control and Prevention, Xining 810007, China; paifu000@hotmail.com

**Keywords:** randomized trial, 24 h urinary sodium excretion, salt reduction, China

## Abstract

Background: Salt intake in China was high and a series of salt reduction measures were accordingly carried out recently. Our study aimed to assess the long-term effect of a scale-up community randomized controlled trial (RCT); Methods: Individuals between the ages of 18 and 75, from six provinces in China, were recruited and randomized into control (*n* = 1347) and intervention (*n* = 1346) groups. A one-year salt reduction intervention was first implemented in the intervention group, followed by a two-year scale-up intervention in both groups. The 24 h urine sample, anthropometric measurement, and knowledge, attitude, and practice (KAP) of salt reduction, as well as lifestyle information, were collected at baseline, after one-year RCT (mid-term evaluation, *n* = 2456), and two-year scale-up intervention (terminal evaluation, *n* = 2267); Results: Both control (351.82 mg/24 h, *p* < 0.001) and intervention (192.84 mg/24 h, *p* = 0.006) groups showed a decrease in 24 h urinary sodium excretion from baseline to terminal evaluation. Except for an increase in 24 h urinary potassium excretion (85.03 mg/24 h, *p* = 0.004) and a decrease in systolic blood pressure (SBP) (2.95 mm Hg, *p* < 0.001) in the intervention group at the mid-term assessment, no statistically significant differences in other indicators were found between two groups. The KAP of salt reduction in two groups was gradually improved; Conclusions: After one-year RCT and two-year scale-up, all participants showed a decreasing trend in 24 h urinary sodium excretion and an increase in salt reduction KAP. The community salt reduction intervention package has the potential for broader application across other regions in China.

## 1. Introduction

Evidence has demonstrated that excessive salt intake can lead to raised blood pressure (BP) and is one of the major risk factors for cardiovascular disease [1,2,3,4]. In China, over 1.5 million deaths were associated with a high-salt diet in 2017, which has emerged as the third-leading risk factor for disability (adjusted for year of age and death) [5].

Reduction in sodium intake is one of the cost-effective ways to improve health and reduce the burden of noncommunicable diseases (NCDs) [6]. The World Health Organization (WHO) recommends a maximum sodium intake of <2000 mg per day (<5 g/d salt) in adults [7]; however, the mean sodium intake in China, based on 24 h urinary sodium, was more than twice the WHO recommendation [8]. In developed countries, about 80% of salt was added by the food industry to processed, restaurant, and fast foods, but the main source (80%) of sodium in the Chinese population was salt added during home cooking or at the table, and this casual use of salt is highly variable and difficult to quantify through dietary surveys [9]. In addition to high salt intake, studies have demonstrated that low potassium intake and a high Na/K ratio also have strong independent dose-response associations with increased blood pressure [10].

The Action on Salt China (ASC) program, which was launched in 2017, aimed to reduce salt intake by conducting a set of targeted interventions in different scenarios [11]. The community Comprehensive Intervention Study (CIS) [12] included in the ASC program was a randomized controlled trial (RCT) designed to evaluate the effectiveness of the community salt reduction intervention package. After one-year RCT, an increase in 24 h urinary potassium excretion and a decrease in systolic blood pressure (SBP) was observed, and knowledge and awareness of salt reduction improved [12]. In order to benefit the general public, the intervention strategies were scaled up in the second and third years, including for those originally randomized to the control group. The purpose of this study was to evaluate the long-term effects of expanding a community-based salt reduction intervention study, which may provide a reference for the future development of salt reduction strategies in China.

## 2. Materials and Methods

### 2.1. Study Design

The study was an RCT followed by a scale-up of intervention in Hebei, Heilongjiang, Jiangxi, Hunan, Sichuan, and Qinghai provinces, which are located in the western, central, and eastern regions of China. At baseline, 48 towns (clusters) were selected from 12 counties (districts) in six provinces. The townships were stratified according to their population size and level of development and randomized into control and intervention groups. The baseline investigation was conducted from October to December 2018. At one year, intervention in the intervention group was started after the baseline survey; the mid-term evaluation (at the end of the RCT) was carried out from November 2019 to January 2020. The two-year scaled-up intervention, which covered both intervention and control groups, was implemented; the terminal evaluation was executed between September and October 2021. A detailed methodology description has been published elsewhere [12].

The research was approved by the Institutional Review Board of the National Center for Chronic and Noncommunicable Disease Control and Prevention, China CDC (201807), and the Queen Mary Ethics of Research Committee (QMERC 2018/16). All participants have submitted written informed consent and can terminate their participation at any time.

### 2.2. Study Participants

Eligible participants were aged 18–75 years old, had lived in their local areas for more than 6 months, and had no plans to relocate. Only one person per family could qualify. Pregnant or breastfeeding women, participants unwilling or unable to collect 24 h urine samples, and patients with serious mental and physical illnesses, which would affect the results of the urine sodium test (such as kidney disease), were all excluded.

### 2.3. Randomization and Masking

After the baseline survey, towns (clusters) stratified by province were randomized 1:1 to intervention and control groups through a computerized randomization sequence. Respondents and local staff responsible for data collection were not aware of the results until the intervention started.

### 2.4. Data Collection

All outcomes were collected at baseline, mid-term, and terminal evaluations for all participants. Data were gathered and managed by a mobile electronic data collection system, which helped standardize the collection procedure by well-trained research staff. A standardized procedure was followed to collect 24 h urine samples. Concentrations of sodium, potassium, and creatinine in urine samples were measured in a uniform laboratory. BP was measured three times by trained local physicians using a validated electronic BP monitor (HEM-7125). Weight, height, and outdoor temperature were measured and collected by calibrated machines. Indicators of salt reduction knowledge, attitude, and practice (KAP), as well as alcohol consumption, physical activity, and history of hypertension and medication use among hypertensive patients, were obtained by face-to-face inquiry by the investigators. Detailed data collection methods are available in other published literature [12].

### 2.5. Intervention

A multifaceted comprehensive intervention strategy for salt reduction was proposed, drawing on evidence from previous studies [2,13,14]. The intervention package, designed by the CIS national project office, was conducted by local governments, county CDC staff, and primary care doctors. Other major stakeholders, such as women’s federations, publicity department, hospitals, schools, and restaurants, were also involved in developing the intervention. All local health educators received training from project staff during a three-day workshop. The intervention included salt reduction lectures, activities, educational materials and supportive tools. The details of the intervention procedures and resources are described in Supplementary Table S1.

The salt reduction lectures consisted of four sessions: salt and health relationships, salt reduction goals, skills for reducing salt intake, and the usage of low-sodium salt substitutes. Each session lasted no less than 40 min and used standardized materials. Lectures are held every three months by trained county CDCs. Salt-reduction-themed activities included salt reduction knowledge competitions in public places, family healthy cooking competitions in communities, and salt-reduction-related handwritten artwork in school. Salt reduction educational materials and supportive tools included posters, brochures, leaflets, signs, and salt-restricted spoons. These materials were distributed to community residents through a variety of activities in public places, schools, and hospitals. Salt-restricting spoons, which were designed to measure salt intake during cooking, were distributed to the family chef. Each spoon contains 2 g of salt, making it convenient for chefs to measure and control the amount used during cooking.

In the first year, interventions were implemented in the intervention group and no additional interventions regarding salt reduction were conducted within the control group. Participants in the control group continued with their standard health management practices. After a one-year evaluation, all interventions were conducted simultaneously in the intervention and control groups during the scale-up period. The scale-up intervention was implemented during the COVID-19 epidemic. To control the pandemic, some regions in China has introduced a number of measures including lockdown. All salt reduction interventions were suspended or postponed during the lockdown period in each locations.

### 2.6. Outcomes

Primary outcomes were the change in salt intake registered in 24 h urinary sodium excretion, 24 h urinary potassium excretion, and BP taken at the baseline, mid-term, and terminal evaluations among the control and intervention groups. The salt intake was calculated by 24 h urinary sodium excretion: salt intake (g/d) = urinary sodium excretion (mg/24 h) × (58.5/23)/1000. Secondary outcomes were variations in salt reduction KAP from the baseline, mid-term, and terminal evaluations in both groups.

### 2.7. Statistical Analysis

In accordance with the findings from other studies [11,12], a sample of 24 cluster pairs (48 towns) with 56 participants per cluster could achieve an 80% capacity to register a difference of 25 mmol/24 h (1.46 g/day salt) between the group means, assuming the SD (standard deviation) was 85.0 and the interclass correlation coefficient (ICC) was 0.080. A two-sided *t*-test of the mean difference was assumed, with a significance level of 0.05. Finally, a total of 2688 individuals were recruited.

Samples were excluded from the data analysis if the urine volume was less than 500 mL, or if the creatinine level was below 4.0 mmol/24 h for women or below 6.0 mmol/24 h for men. If the 24 h urine collections were excluded from either baseline or evaluation surveys, we used only the complete one. In cases where the duration of urinary collection deviated from precisely 24 h, an adjusted 24 h urinary volume was determined by dividing the actual urine volume by the actual collection period and then multiplying the result by 24. The 24 h excretions of sodium and potassium were calculated by multiplying the urine volume by concentration. For BP, the mean of the last two measurements was included in the analysis. Hypertension was diagnosed based on a mean systolic blood pressure (SBP) of 140 mmHg or higher, and/or a mean diastolic blood pressure (DBP) of 90 mmHg or higher, and/or self-reported use of anti-hypertensive drugs in two weeks before the survey [15].

The data were analyzed utilizing the intention-to-treat (ITT) methodology. Linear mixed models with a random intercept were employed to evaluate the intervention effect on primary outcomes, considering three level clusters: individual-level data were nested within village-level data and the village-level data were nested in county-level. The intervention effects on KAP were analyzed by using the GLIMMIX model (Generalized Linear Mixed Model) which are suitable for the analysis of non-normally distributed response variable data such as binary categorical variables [16]. The random intercept for the model were at two levels (individual-level and village-level). The fixed effect variables included groups (intervention and control), time periods (baseline, mid-term, and terminal evaluations), and interaction between time and group. The interaction term between time and group indicated the differential change in outcome measurements from baseline to mid-term and terminal evaluations between the intervention and control groups. Potential confounding variables including sex (male = 0; female = 1), age group (<40 = 1, 40~60 = 2, ≥60 = 3), body mass index (BMI), and were adjusted in the linear mixed models. For BP, adjusting factors also included outdoor temperature, alcohol drinking status (non-drinker, occasional drinker, or regular drinker), and physical activity (moderate physical activity for at least 30 min at least three times per week or not).

Furthermore, we conducted two sensitivity analyses on urinary outcome measures to assess the robustness of the conclusions derived from the primary analysis. The first analysis included all the participants who participated in the three surveys, and it is possible that the 24 h urine samples were incompletely collected. The second analysis only encompassed participants who completed all three surveys and also collected complete urine samples in each survey (named as completers).

The statistical analysis was conducted using SAS (version 9.4). Twenty-four-hour urine and blood pressure measurements were reported as means, SDs, and 95% confidence intervals (CIs). Categorical variables were presented as frequencies and percentages, along with odds ratios (ORs) and 95% CIs where applicable. All statistical analyses were conducted using a two-tailed approach, with a significance level set at *p* < 0.05.

## 3. Results

### 3.1. Characteristics of Participants

**Figure 1:**
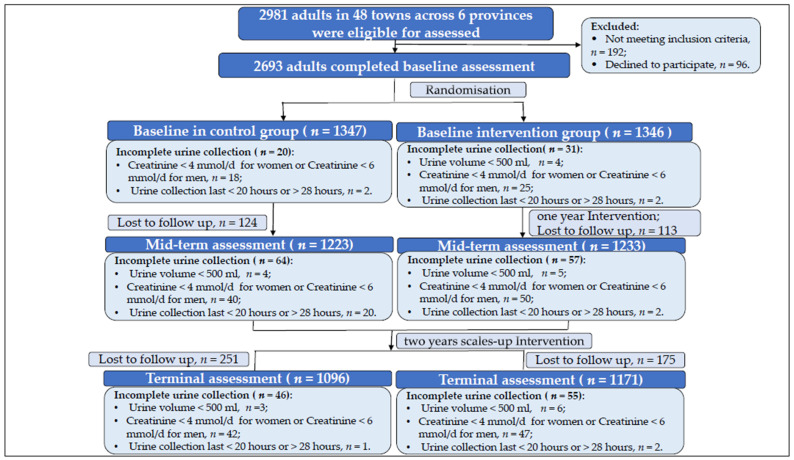
Flowchart of individuals in the baseline and two evaluation surveys.

Forty-eight towns (24 in the control group and 24 in the intervention group), across six provinces, were followed up at first-year and third-year after intervention was implemented. At baseline, 2981 adults were recruited, and 2693 individuals completed the survey. After randomization, 1346 individuals in 24 towns were assigned to the intervention group, while 1347 individuals in 24 towns were designated to the control group. During the mid-term evaluation (n = 2456), 237 out of 2693 participants (8.8%; 114 in the intervention group and 123 in the control group) were lost to follow-up due to relocation or inability to attend the survey. Upon excluding 121 incomplete urine samples, 2335 samples were eligible for the 24 h urine analysis. At the terminal investigation (n = 2267), 426 out of 2693 participants (15.8%; 251 in the intervention group and 175 in the control group) were lost, and 2166 qualified 24 h urine samples were included in the analysis. The numbers of respondents at baseline and two assessment surveys are shown in Figure 1.

The characteristics of participants in the baseline and two follow-up surveys are showed in Table 1. Hypertension self-reporting and treatment in self-reported hypertensives improved after the intervention. Other characteristics of individuals were similar in the three investigations.

### 3.2. Primary Outcome

Table 2 and Table 3 show the results and changes in 24 h urinary sodium excretion, other urinary measurements, and BP, with adjustment for confounders in the baseline and two subsequent follow-ups. The 24 h urinary sodium excretion at baseline, mid-term, and terminal evaluations were 4510.6 mg/24 h (11.5 g/d of salt), 4430.9 mg/24 h (11.3 g/d of salt), and 4251.4 mg/24 h (10.8 g/d of salt) in the control group, compared to 4363.0 mg/24 h (11.1 g/d of salt), 4349.8 mg/24 h (11.1 g/d of salt), and 4196.2 mg/24 h (10.6 g/d of salt) in the intervention group, respectively (Table 2). The comparative analysis of 24 h urinary sodium excretion between the control and intervention groups in the baseline and terminal evaluations showed no significant difference (158.98 mg/24 h, *p* = 0.11) (Table 3); however, a statistically significant decrease in the 24 h urinary sodium excretion within both control and intervention groups was observed. Changes in 24 h urinary sodium excretion from the covariates-adjusted mixed model were −351.82 mg/24 h (*p* < 0.001) in the control group and −192.84 mg/24 h (*p* = 0.006) in the intervention group.

Table 2 also shows that the 24 h urinary potassium excretion at baseline, mid-term, and terminal evaluations were 1584.6 mg/24 h, 1519.9 mg/24 h, and 1510.4 mg/24 h in the control group, and 1546.2 mg/24 h, 1573.4 mg/24 h, and 1490.0 mg/24 h in the intervention group, respectively. No statistically significant difference in 24 h urinary potassium excretion was seen between the two groups in the terminal evaluation (−0.19 mg/24 h, *p* = 0.98) (Table 3); however, 24 h urinary potassium excretion was significantly decreased from the baseline to the terminal evaluation within both control and intervention groups. The changes based on the covariates-adjusted mixed model were −77.30 mg/24 h (*p* = 0.002) in the control group and −79.37 mg/24 h (*p* = 0.004) in the intervention group. There was no statistically significant difference in the sodium–potassium ratio between the intervention group and control group when comparing baseline and terminal evaluations (*p* > 0.05) (Table 3).

The systolic and diastolic BP at baseline were 125.6 mmHg and 79.0 mmHg in the control group, compared to 127.2 mmHg and 80.1 mmHg in the intervention group. At the terminal evaluation, the systolic and diastolic BP were 128.1 mmHg and 79.5 mmHg in the control group, compared to 129.0 mmHg and 80.1 mmHg in the intervention group (Table 2). No significant effect was observed on systolic or diastolic BP when comparing the intervention group to the control group during the final evaluation (*p* = 0.05) (Table 3); However, the SBP in the intervention group was 2.95 mmHg lower than the control group during the mid-term survey (*p* < 0.001). The results of salt intake, 24 h urinary sodium excretion, additional urine measurements, and BP changes without adjustment for confounding variables are shown in Supplementary Table S2, which is similar to Table 3.

Supplementary Tables S3 and S4 presented the results of the sensitivity analyses. When comparing the intervention group to the control group, the intervention effect remained consistent with the primary outcomes.

### 3.3. Secondary Outcomes

Table 4 and Table 5 show the results and changes in KAP in baseline and two evaluation surveys. Comparing the baseline with the terminal evaluation, a significant intervention effect was observed regarding levels of knowledge of the recommended salt intake (from 24.5% to 39.2% in the control group vs. from 17.3% to 68.9% in the intervention group, OR = 5.53, *p* < 0.001), having heard of low-sodium salt substitutes (from 25.4% to 45.0% in the control group vs. from 27.6% to 58.5% in the intervention group, OR = 1.58, *p* < 0.001), ability to identify salt content on food labels (from 42.8% to 50.5% in the control group vs. from 37.4% to 62.0% in the intervention group, OR = 2.14, *p* < 0.001), and willingness to choose a low-sodium diet (from 79.8% to 85.8% in the control group vs. from 79.3% to 94.0% in the intervention group, OR = 2.75, *p* < 0.001). In the intervention group, there was a statistically significant increase in the proportion of respondents who preferred a less salty flavor (27.2% in baseline vs. 35.3% in terminal, OR = 1.44, *p* = 0.001), using low-sodium salt substitutes (35.0% in baseline vs. 41.4% in terminal, OR = 1.34, *p* = 0.04), and consuming processed food once per week or less (58.5% in baseline vs. 62.8% in terminal, OR = 1.18, *p* = 0.04). However, no statistically significant difference was found in these indicators between the intervention and control groups (*p* > 0.05).

## 4. Discussion

Our study was a long-term, town-level comprehensive intervention study to reduce salt intake in China, with three years of intervention (first year in the intervention group and second–third years in the intervention and control groups) and follow-up across the whole population. Although there was no significant difference in salt intake/24 h urinary sodium excretion between the intervention and control groups after the scale-up intervention, a significant downward trend in 24 h urinary sodium excretion was observed within the two groups. Levels of salt reduction knowledge and attitude were increased, and the salt reduction behavior in the intervention group improved gradually on the basis of the first-year intervention.

Excessive sodium intake has been associated with increased arterial stiffness and endothelial dysfunction and decreased calcium level, resulting in an increase in peripheral vascular resistance, which could lead to high BP and cardiovascular risk [17,18]. A population-level multifaceted comprehensive intervention strategy for salt reduction can reduce dietary salt intake [19,20]. A systematic review of the global salt reduction initiative shows that, as of 2019, 96 countries or territories have adopted different structural or regulatory approaches to salt reduction. Although most countries are experiencing a downward trend in salt intake, none have yet met the targeted 30% reduction in salt intake from baseline [21]. A series of regional multifaceted salt reduction programs was implemented in China in recent years; for example, a five-year salt reduction intervention in Shandong Province, with interventions such as media campaigns, distribution of scaled spoons, promotion of low-sodium products in shopping malls and restaurants, and salt reduction education at home and in schools, showed that 24 h urinary sodium excretion, SBP and DBP decreased among all participants (1325 mg/d, 1.8 mmHg, and 3.1 mm Hg, respectively) [13]. The results of an app-based salt reduction education program in primary schools, using a child-to-parent approach, which is a part of the same ASC project as our study, showed that, although the salt intake among primary school children did not change, the salt intake and SBP of parents decreased by 0.82 g/d and 1.64 mm Hg, respectively [22]. In addition to these studies, the Chinese government has introduced a series of salt reduction policies, such as China Healthy lifestyle for All and Healthy China [23,24], and the average amount of cooking salt consumed by Chinese residents aged 18 years and above per person per day was declining, from 10.5 g in 2015 to 9.3 g in 2020 [25,26]. Therefore, the control population in our study also received usual health management activities, which included salt reduction components, and showed some decrease in 24 h urinary sodium in the first year. In the terminal evaluation, a significantly greater decrease in 24 h urinary sodium excretion was observed in both intervention and control groups. The effects of salt reduction interventions are sustained. We also found that the duration of salt reduction intervention was an important influencing factor on the effectiveness of the intervention. Due to differing baseline characteristics between the intervention and control groups (the intervention group exhibited higher prevalence of hypertension, lower education level, and, most significantly, lower 24 h urinary sodium excretion compared with the control group at the baseline survey), more effort and time might be needed to achieve positive results. Some studies have indicated that [27,28], after experiencing the outbreak of COVID-19, respondents experiencing greater psychological impact were more inclined to increase their consumption of salt, fried foods, and sugary foods. Our terminal evaluation, which was implemented during the epidemic, showed that 24 h urinary sodium excretion maintained on a downward trend in both groups. The community salt reduction package played a positive role.

In addition to the 24 h urinary sodium excretion, we found that urinary potassium increased in the intervention group after the one-year intervention; however, there was a statistically significant decrease in both groups during the terminal investigation. The reason may be related to the timing of the scaled-up interventions (2020 and 2021) falling within COVID-19 pandemic period in China. Due to the lockdown and others measures to control the epidemic, respondents were restricted from going out shopping and reduced their intake of fresh fruits and vegetables, which led to a general decrease in 24 h urinary potassium excretion [29]. As a result of multifactorial influences, such as decreased 24 h urinary potassium excretion after the scaled-up intervention, it is not surprising that there was no significant difference in BP between the intervention group and the control group.

In terms of KAP, the intervention group showed better improvements in knowledge and attitudes than the control group after scale-up intervention. In-group comparisons showed all KAP indicators improved in the intervention group after three years of intervention but, in the control group, only knowledge and some attitude indicators showed improvement after two years of scale-up intervention, with no significant changes in behavior. It suggested that one- or two-year salt reduction interventions may be able to increase the knowledge of salt reduction in community populations and create a willingness to reduce salt, but are not sufficient to change the habits of the study populations or make them take action to reduce salt intake [30]. Most studies with positive outcomes in salt reduction interventions for community populations through health education tools have lasted more than one year [13,31,32]. Therefore, sustained publicity campaigns on salt reduction should be conducted to help people to acquire knowledge and to change their attitudes, which could be translated into actions gradually.

Strengths of our study include that it was a comprehensive large-scale community-based salt reduction intervention study covering six provinces across the eastern, central, and western regions of China, and all respondents were followed up for three years (loss of follow-up rate = 15.8%). A set of salt reduction intervention packages for community populations was developed and disseminated to the population. In order to ensure data quality, a mobile electronic data collection system was utilized to collect and manage data. Blind methods are used in the laboratory testing of urine samples to avoid potential bias.

Our study had several limitations. Firstly, the scaled-up intervention coincided with the COVID-19 pandemic, which led to significant changes in people’s diet and lifestyles and may have had an impact on the results of the study. Secondly, single 24 h urine samples were collected by the participants, and the results, which were affected by diet on the day of collection, may not fully reflect the salt intake level. Thirdly, other indicators affecting 24 h urinary sodium excretion, such as urinary calcium, taking certain medications, e.g., sodium–glucose cotransporter 2 inhibitors or recombinase-aid amplification inhibitors, were not collected.

## 5. Conclusions

This study demonstrated that, after three years of salt reduction intervention in the intervention group and two years of scale-up intervention in the control group, there was a significant decrease in 24 h urinary sodium excretion in two groups. SBP decreased in the intervention group compared to the control group after one-year intervention; however, no statistically significant differences were observed in the terminal assessment between the two groups. Additionally, salt reduction KAP gradually improved in both groups. The community salt reduction intervention package has the potential for broader application across other regions in China. Moreover, implementing various public health policies such as the Health China Salt Reduction Initiative is also crucial to achieving the goal of a 20% reduction of daily salt intake as per the Healthy China 2030 plan.

## Figures and Tables

**Table 1:** The characteristics of individuals in three investigations.

Characteristics	Baseline		Mid-Term Evaluation		Terminal Evaluation	
	Control (N = 1347)	Intervention (N = 1346)	Control (N = 1223)	Intervention (N = 1233)	Control (N = 1096)	Intervention (N = 1171)
Outdoor temperature, Mean (SD)	7.2 (9.2)	8.1 (7.7)	4.2 (9.1)	5.0 (11.7)	7.6 (10.9)	9.60 (7.3)
Age (years), Mean (SD)	47.2 (13.0)	48.8 (12.6)	48.7 (12.9)	50.2 (12.5)	50.4 (13.0)	51.8 (12.6)
Males, n (%)	667 (49.5)	667 (49.6)	600 (48.6)	608 (54.1)	532 (48.5)	595 (50.9)
Education level (n, %)						
Primary school or lower	540 (40.1)	596 (44.3)	498 (40.4)	489 (43.5)	432 (39.4)	498 (42.6)
Secondary school	536 (39.8)	503 (37.4)	481 (39.0)	417 (37.1)	424 (38.7)	444 (38.0)
High school	159 (11.8)	161 (12.0)	152 (12.3)	149 (13.3)	127 (11.6)	141 (12.1)
University or college	112 (8.3)	86 (6.4)	102 (8.3)	68 (6.1)	113 (10.3)	87 (7.4)
BMI (kg/m^2^), Mean (SD)	24.7 (3.7)	24.8 (3.5)	24.8 (3.7)	24.9 (3.5)	24.9 (3.6)	25.1 (3.6)
Physical activity: active (n, %)	567 (42.1)	519 (38.6)	499 (40.5)	454 (40.4)	464 (42.3)	477 (40.7)
Self-reported hypertension (n, %)	233 (17.4)	300 (22.3)	227 (18.4)	274 (24.4)	216 (19.7)	280 (23.9)
BP treatment in self-reported hypertensives (n, %)	157 (67.3)	247 (82.3)	172 (75.8)	239 (87.2)	180 (83.3)	240 (85.7)

Abbreviations: BMI, body mass index; BP, Blood Pressure.

**Table 2:** Results of 24 h urinary sodium excretion, other urinary indicators, and blood pressure in three surveys.

Outcomes	Control			Intervention		
	Baseline	Mid-Term	Terminal	Baseline	Mid-Term	Terminal
	N,	N,	N,	N,	N,	N,
	Mean (SD)	Mean (SD)	Mean (SD)	Mean (SD)	Mean (SD)	Mean (SD)
Salt intake (g/d)	1327	1159	1050	1315	1176	1116
	11.5 (4.8)	11.3 (4.7)	10.8 (4.6)	11.1 (4.5)	11.1 (4.8)	10.6 (4.5)
Urinary sodium (mg/24 h)	1327	1159	1050	1315	1176	1116
	4510.6 (1867.0)	4430.9 (1860.8)	4251.4 (1805.9)	4363.0 (1770.9)	4349.8 (1894.1)	4169.2 (1759.7)
Urinary potassium (mg/24 h)	1327	1159	1050	1315	1176	1116
	1584.6 (654.0)	1519.9 (589.4)	1510.4 (636.3)	1546.2 (637.0)	1573.4 (634.6)	1490.0 (625.5)
Sodium-to-potassium ratio	1327	1159	1050	1315	1176	1116
	5.2 (2.3)	5.3 (2.3)	5.2 (2.2)	5.1 (2.1)	5.0 (2.2)	5.2 (2.2)
Systolic blood pressure (mmHg)	1347	1223	1096	1346	1233	1171
	125.6 (19.3)	128.0 (20.0)	128.1 (19.9)	127.2 (19.3)	126.8 (18.8)	129.0 (19.4)
Diastolic blood pressure (mmHg)	1347	1223	1096	1346	1233	1171
	79.0 (11.8)	79.6 (12.3)	79.5 (12.1)	80.1 (11.4)	80.0 (11.5)	80.1 (11.7)

**Table 3:** Changes in 24 h urinary sodium excretion, other urinary indicators, and blood pressure from the covariates-adjusted mixed linear model.

Outcomes	Control		Intervention		Adjusted Difference in Change * (Intervention vs. Control)	
	Difference (95% CI)	*p*-Value	Difference (95% CI)	*p*-Value	Difference (95% CI)	*p*-Value
Salt intake (g/d)						
Mid-term vs. Baseline	−0.23 (−0.53 to 0.06)	0.09	−0.02 (−0.31 to 0.27)	0.86	0.22 (−0.21 to 0.64)	0.31
Terminal vs. Mid-term	−0.65 (−1.02 to −0.29)	<0.001	−0.46 (−0.82 to −0.11)	0.01	0.19 (−0.32 to 0.70)	0.46
Terminal vs. Baseline	−0.89 (−1.25 to −0.54)	<0.001	−0.49 (−0.84 to −0.14)	0.006	0.40 (−0.09 to 0.90)	0.11
Urinary sodium (mg/24 h)						
Mid-term vs. Baseline	−91.99 (−208.47 to 24.50)	0.12	−6.40 (−122.63 to 109.83)	0.86	85.58 (−78.82 to 249.99)	0.31
Terminal vs. Mid-term	−259.83 (−401.66 to −116.95)	<0.001	−184.44 (323.83 to 40.71)	0.01	73.40 (−123.18 to 273.20)	0.46
Terminal vs. Baseline	−351.82 (−491.17 to −212.48)	<0.001	−192.84 (−329.74 to −55.94)	0.006	158.98 (−35.90 to 353.86)	0.11
Urinary potassium (mg/24 h)						
Mid-term vs. Baseline	−64.45 (−105.30 to −23.60)	0.003	20.58 (−20.18 to 61.34)	0.28	85.03 (27.38 to 142.68)	0.004
Terminal vs. Mid-term	−12.85 (−64.27 to 38.56)	0.62	−99.95 (−149.93 to −48.40)	<0.001	−86.50 (−158.42 to −14.57)	0.02
Terminal vs. Baseline	−77.30 (−127.55 to −26.80)	0.002	−79.37 (−121.81 to −29.92)	0.004	−0.19 (−70.60 to 70.22)	0.98
Sodium-to-potassium ratio						
Mid-term vs. Baseline	0.09 (−0.05 to 0.24)	0.33	−0.07 (−0.22 to 0.07)	0.33	−0.17 (−0.37 to 0.04)	0.12
Terminal vs. Mid-term	−0.22 (−0.39 to −0.04)	0.008	0.15 (−0.02 to 0.33)	0.08	0.37 (0.12 to 0.62)	0.003
Terminal vs. Baseline	−0.13 (−0.30 to 0.05)	0.02	0.08 (−0.09 to 0.25)	0.34	0.21 (−0.04 to 0.45)	0.09
Systolic blood pressure (mmHg)						
Mid-term vs. Baseline	0.71 (−0.21 to 1.63)	0.17	−2.24 (−3.21 to −1.27)	<0.001	−2.95 (−4.08 to −1.83)	<0.001
Terminal vs. Mid-term	0.81 (−0.31 to 2.03)	0.22	2.26 (1.08 to 3.44)	<0.001	1.45 (−0.02 to 2.96)	0.05
Terminal vs. Baseline	1.52 (0.85 to 2.19)	<0.001	0.02 (−0.14 to 1.18)	0.67	−1.50 (−2.96 to 0.04)	0.05
Diastolic blood pressure (mmHg)						
Mid-term vs. Baseline	−0.89 (−1.49 to −0.29)	0.82	−1.34 (−1.91 to −0.71)	0.39	−0.45 (−1.17 to 0.27)	0.22
Terminal vs. Mid-term	1.47 (−0.15 to 3.09)	0.06	0.75 (−0.41 to 1.09)	0.66	−0.92 (−1.22 to 0.30)	0.13
Terminal vs. Baseline	0.58 (−0.02 to 1.14)	0.08	−0.54 (−1.62 to 0.54)	0.45	−1.02 (−2.46 to 1.44)	0.05

* Model-based adjustments for urinary outcomes account for age categories, gender, and BMI at baseline and two follow-up surveys. Model-based adjustments for systolic and diastolic blood pressure are further adjusted for outdoor temperature, physical activity, and alcohol drinking status.

**Table 4:** Results for salt reduction KAP in baseline, mid-term, and terminal evaluations.

	Control			Intervention		
	Baseline N (%)	Mid-Term N (%)	Terminal N (%)	Baseline N (%)	Mid-Term N (%)	Terminal N (%)
Knowledge						
Knowledge of the recommended salt intake	330 (24.5)	375 (30.4)	430 (39.2)	233 (17.3)	889 (71.6)	808 (68.9)
Having heard of low-sodium salt substitutes	342 (25.4)	458 (37.1)	493 (45.0)	371 (27.6)	725 (58.4)	685 (58.5)
Having ability to identify salt content on food labels	577 (42.8)	505 (40.9)	553 (50.5)	504 (37.4)	784 (63.2)	727 (62.0)
Attitude						
Willingness to choose a low-sodium diet	1075 (79.8)	991 (80.3)	940 (85.8)	1067 (79.3)	1099 (88.6)	1103 (94.0)
Preferring a less salty flavor	380 (28.2)	372 (30.2)	354 (32.3)	366 (27.2)	369 (29.7)	414 (35.3)
Practice						
Using low-sodium salt substitutes ^a^	109 (31.9)	133 (29.0)	150 (30.1)	130 (35.0)	239 (33.0)	284 (41.4)
Consuming processed food once per week or less	746 (55.4)	714 (57.9)	596 (54.4)	788 (58.5)	814 (65.6)	736 (62.8)

^a^ Questions of low-sodium salt substitutes use surveyed among individuals who have heard about it.

**Table 5:** Results for salt reduction KAP in baseline, mid-term, and terminal evaluations from covariates-adjusted mixed line model.

	Control		Intervention		Adjusted Difference in Change * (Intervention vs. Control)	
	Difference (95% CI)	*p*-Value	Difference (95% CI)	*p*-Value	Difference (95% CI)	*p*-Value
Knowledge						
Knowledge of the recommended salt intake						
Mid-term vs. Baseline	1.38 (1.16 to 1.64)	0.004	12.99 (10.73 to 15.72)	<0.001	9.43 (7.28 to 12.21)	<0.001
Terminal vs. Mid-term	1.52 (1.28 to 1.81)	<0.001	0.89 (0.75 to 1.06)	0.19	0.58 (0.46 to 0.75)	<0.001
Terminal vs. Baseline	2.09 (1.75 to 2.50)	<0.001	11.55 (9.55 to 13.98)	<0.001	5.53 (4.27 to 7.16)	<0.001
Having heard about low-sodium salt substitutes						
Mid-term vs. Baseline	1.81 (1.52 to 2.15)	<0.001	3.99 (3.37 to 4.72)	<0.001	2.20 (1.73 to 2.80)	<0.001
Terminal vs. Mid-term	1.45 (1.22 to 1.71)	<0.001	1.04 (0.88 to 1.23)	0.64	0.72 (0.57 to 0.91)	<0.001
Terminal vs. Baseline	2.62 (2.20 to 3.12)	<0.001	4.14 (3.49 to 4.91)	<0.001	1.58 (1.24 to 2.02)	<0.001
Having ability to identify salt content on food labels						
Mid-term vs. Baseline	0.97 (0.83 to 1.15)	0.70	3.40 (2.87 to 4.03)	<0.001	3.50 (2.76 to 4.43)	<0.001
Terminal vs. Mid-term	1.59 (1.34 to 1.89)	<0.001	1.00 (0.83 to 1.18)	0.93	0.62 (0.49 to 0.80)	<0.001
Terminal vs. Baseline	1.54 (1.30 to 1.82)	<0.001	3.30 (2.78 to 3.91)	<0.001	2.14 (1.69 to 2.72)	<0.001
Attitude						
Willingness to choose a low-sodium diet						
Mid-term vs. Baseline	1.02 (0.84 to 1.24)	0.08	2.03 (1.63 to 2.53)	<0.001	1.99 (1.48 to 2.66)	<0.001
Terminal vs. Mid-term	1.48 (1.19 to 1.85)	<0.001	2.06 (1.52 to 2.78)	<0.001	1.39 (0.95 to 2.01)	0.09
Terminal vs. Baseline	1.52 (1.22 to 1.89)	<0.001	4.18 (3.17 to 5.12)	<0.001	2.75 (1.94 to 3.92)	<0.001
Preferring a less salty flavor						
Mid-term vs. Baseline	1.08 (0.91 to 1.28)	0.43	1.12 (0.94 to 1.33)	0.24	1.04 (0.81 to 1.32)	0.77
Terminal vs. Mid-term	1.10 (0.90 to 1.33)	0.33	1.29 (1.07 to 1.56)	0.01	1.17 (0.89 to 1.54)	0.23
Terminal vs. Baseline	1.18 (0.91 to 1.43)	0.09	1.44 (1.19 to 1.73)	0.001	1.22 (0.93 to 1.59)	0.14
Practice						
Using low-sodium salt substitutes						
Mid-term vs. Baseline	0.91 (0.67 to 1.24)	0.50	1.01 (0.77 to 1.32)	0.64	1.11 (0.74 to 1.67)	0.62
Terminal vs. Mid-term	1.05 (0.77 to 1.42)	0.76	1.43 (1.13 to 1.82)	0.006	1.37 (0.93 to 2.02)	0.11
Terminal vs. Baseline	0.94 (0.68 to 1.30)	0.69	1.34 (1.01 to 1.79)	0.04	1.43 (0.92 to 2.21)	0.10
Consuming processed food once per week or less						
Mid-term vs. Baseline	1.10 (0.94 to 1.29)	0.23	1.34 (1.14 to 1.58)	<0.001	1.22 (0.97 to 1.52)	0.09
Terminal vs. Mid-term	0.86 (0.73 to 1.01)	0.07	0.88 (0.74 to 1.04)	0.13	1.02 (0.81 to 1.29)	0.86
Terminal vs. Baseline	0.95 (0.81 to 1.11)	0.51	1.18 (1.01 to 1.38)	0.04	1.24 (0.99 to 1.56)	0.06

* Model-based changes for KAP outcomes are adjusted for age categories, gender, education level, and BMI at baseline and two follow-up surveys.

## Data Availability

Data are available upon request.

## Supplementary Materials

The following supporting information can be downloaded at: https://www.mdpi.com/article/10.3390/nu16234092/s1, Table S1: Composition of the intervention package and implementation plan; Table S2: Changes in 24 h urinary sodium excretion, other urinary indicators, and blood pressure without adjusting confounders; Table S3: Sensitivity analysis for 24 h urinary sodium and potassium excretion; Table S4: Sensitivity analysis for 24 h urinary sodium and potassium excretion changes from covariates-adjusted mixed linear model.
