# Supplementary material for: Long-Term Effects of a Comprehensive Intervention Strategy for Salt Reduction in China: Scale-Up of a Cluster Randomized Controlled Trial
Source: Nutrients. 2024 Nov 27;16(23):4092. doi: 10.3390/nu16234092 (PMC11643943; doi:10.3390/nu16234092)
Supplement: Supplementary file 1 [file nutrients-16-04092-s001.zip › nutrients-3312770-supplementary.pdf]

Supplementary Table S1 Composition of the intervention package and implementation plan

Location	Intervention tools and content	Frequency	Implementers	Target population
Public Place (such as public square, parks and buses)	Salt reduction posters, brochures, leaflets signs and video	At least two publicity days or important holidays a year	County CDCs	Community residents
	Mass culture and publicity activities (such as knowledge competition, family healthy cooking competition)	At least once a year	County governments; Women federations and other major stakeholders	Community residents
	Wechat public account	At least four times a year	County CDCs	Young and mid-aged people
Primary Health Center	Training for all the primary healthcare providers	At least twice a year	County CDCs	Community residents
	Salt reduction lectures and activities	Every three months a year	County CDCs	Community residents
	Publicizing knowledge and tips of salt reduction	During the routine outpatient visits	Primary care doctors	Community residents
Community	Salt-restricting spoons	At least twice a year	County CDCs	Family chef
	Salt Reduction Broadcasting	Every month	Village doctor	Villagers
	Salt reduction posters, brochures, leaflets signs	At least twice a year	Village doctor	Community residents
School	Salt and health training activities	At least twice a year	County CDCs	Teachers and students' parents
	Salt reduction education courses	At least once per month	Teachers	Students
	Public activities (such as production of salt reduction related handwritten artwork)	At least twice a year	Teachers	Students
	Publicity posters on bulletin boards or school canteens	The whole year	School administrators	Teachers and students
Restaurants	Videos, posters and table displays	The whole year	County CDCs	Customers
	Standardized training on how to reduce the amount of salt during cooking and how to guide customers to choose lower salt dishes	At least four times a year	County CDCs	Chef and waiters
	Lower salt dishes are marked on the menu	The whole year	Restaurant managers	Customers

Supplementary Table S2 Changes of 24-h urinary sodium excretion, other urinary indicators and blood pressure without adjusting confounders

Outcomes	Control		Intervention		Difference in change *(intervention vs control)*	
	Difference (*95% CI*)	*P* value	Difference (*95% CI*)	*P* value	Difference (*95% CI*)	*P* value
**Salt intake (g/day)**						
Mid-term vs Baseline	-0.26(-0.56 to 0.04)	0.090	-0.06(-0.36 to 0.24)	0.702	0.20(-0.22 to 0.62)	0.354
Terminal vs Mid-term	-0.66(-1.03 to -0.30)	<0.001	-0.43(-0.78 to -0.07)	0.019	0.24(-0.27 to 0.75)	0.364
Terminal vs Baseline	-0.92(-1.28 to -0.57)	<0.001	-0.49(-0.84 to -0.14)	0.006	0.43(-0.06 to 0.93)	0.086
**Urinary potassium (mg/24 h)**						
Mid-term vs Baseline	-63.74(-105.83 to -21.64)	0.003	23.05(-18.90 to 64.99)	0.281	86.79(27.36 to 146.22)	0.004
Terminal vs Mid-term	-8.54(-59.87 to 42.78)	0.744	-94.71(-145.01 to -44.42)	<0.001	-86.17(-158.03 to -14.31)	0.019
Terminal vs Baseline	-72.28(-122.49 to -22.08)	0.005	-71.66(-120.93 to -22.40)	0.004	0.62(-69.72 to 70.95)	0.986
**Sodium-to-potassium ratio**						
Mid-term vs Baseline	0.07(-0.07 to 0.21)	0.327	-0.10(-0.24 to 0.05)	0.183	-0.17(-0.38 to 0.03)	0.103
Terminal vs Mid-term	-0.24(-0.42 to -0.06)	0.008	0.15(-0.02 to 0.33)	0.090	0.40(0.14 to 0.65)	0.002
Terminal vs Baseline	-0.17(-0.35 to 0.01)	0.058	0.05(-0.12 to 0.23)	0.546	0.22(-0.02 to 0.47)	0.075
**Systolic blood pressure (mmHg)**						
Mid-term vs Baseline	2.29(1.45 to 3.13)	<0.001	-0.58(-1.42 to 0.25)	0.171	-2.87(-4.05 to -1.69)	<0.001
Terminal vs Mid-term	0.55(-0.53 to 1.63)	0.216	1.96(0.90 to 3.02)	<0.001	1.42(-0.10 to 2.93)	0.067
Terminal vs Baseline	2.84(1.77 to 3.90)	<0.001	1.38(0.34 to 2.42)	0.001	-1.46(-2.95 to 0.04)	0.056

**Diastolic blood pressure (mmHg)**						
Mid-term vs Baseline	0.06(-0.47 to 0.59)	0.817	-0.10(-1.01 to 0.82)	0.837	-0.20(-1.49 to 1.08)	0.756
Terminal vs Mid-term	0.81(-0.15 to 1.77)	0.097	-0.20(-1.14 to -0.74)	0.676	-1.01(-2.36 to -0.33)	0.139
Terminal vs Baseline	0.92(-0.02 to 1.86)	0.055	-0.30(-1.22 to 0.62)	0.530	-1.22(-2.54 to 1.00)	0.070

Supplementary Table S3 Sensitivity analysis for 24-h urinary sodium and potassium excretion

Outcomes	Control						Intervention					
	Baseline		Mid-term		Terminal		Baseline		Mid-term		Terminal	
	N	Mean(SD)	N	Mean(SD)	N	Mean(SD)	N,	Mean(SD)	N,	Mean(SD)	N,	Mean(SD)
**Including possible incomplete 24-h urine samples**												
Urinary sodium (mg/24 h)	13<br>47	4488.1 (1883.3)	1223	4346.3 (1877.3)	1095	4172.8 (1848.4)	1346	4328.1 (1813.0)	1233	4260.6 (1915.5)	117<br>0	4117.7 (1782.9)
Urinary potassium (mg/24 h)	13<br>47	1575.9 (658.3)	1223	1494.1 (596.3)	1095	1483.9 (652.1)	1346	1531.5 (646.2)	1233	1536.4 (648.8)	117<br>0	1455.1 (637.2)
**Completer**												
Urinary sodium (mg/24 h)	93<br>6	4606.1 (1930.4)	936	4461.5 (1879.2)	936	4292.7 (1816.2)	996	4409.7 (1769.2)	996	4371.8 (1849.0)	996	4230.2 (1769.0)
Urinary potassium (mg/24 h)	93<br>6	1601.7 (649.1)	936	1544.5 (598.7)	936	1523.3 (641.6)	996	1579.9 (618.0)	996	1587.6 (641.8)	996	1512.8 (624.1)

Supplementary Table S4 Sensitivity analysis for 24-h urinary sodium and potassium excretion changes from covariates-adjusted mixed linear model

Outcomes	Control		Intervention		Difference in change *(intervention vs control)*	
	Difference (*95% CI*)	*P* value	Difference (*95% CI*)	*P* value	Difference (*95% CI*)	*P* value
**Including possible incomplete 24-h urine samples**						
Urinary sodium (mg/24 h)						
Mid-term vs Baseline	-144.41(-261.17 to -27.12)	0.016	-63.49(-180.61 to 53.64)	0.288	80.93(-84.74 to 246.59)	0.330
Terminal vs Mid-term	-258.50(-400.05 to -116.96)	0.0003	-171.8(-311.01 to -32.57)	0.016	86.71(-111.68 to 285.10)	0.392
Terminal vs Baseline	-402.91(-542.37 to -263.46)	<0.001	-235.28(-372.35 to -98.21)	0.008	167.64(-27.47 to 362.74)	0.092
Urinary potassium (mg/24 h)						
Mid-term vs Baseline	-84.19(-125.93 to -42.45)	<0.001	-0.70(-42.37 to 40.98)	0.974	83.49(25.54 to 142.44)	0.005
Terminal vs Mid-term	-12.67(-63.62 to 38.28)	0.626	-99.15(-149.24 to -49.05)	<0.001	-86.48(-157.87 to -15.08)	0.019
Terminal vs Baseline	-96.86(-147.07 to -46.64)	<0.001	-99.84(-149.17 to -50.52)	<0.001	-2.99(-73.21 to 67.23)	0.986
**Completer**						
Urinary sodium (mg/24 h)						
Mid-term vs Baseline	-27.04(-157.62 to 103.54)	0.685	-140.82(-275.43 to -6.21)	0.040	-113.78(-301.25 to 73.69)	0.234
Terminal vs Mid-term	-159.19(-304.16 to -14.22)	0.031	-199.10(-347.63 to 50.57)	0.008	-39.90(247.26 to 167.45)	0.706
Terminal vs Baseline	-186.23(-331.36 to 41.10)	0.011	-339.92(-488.62 to -191.21)	<0.001	-153.69(-361.01 to 53.64)	0.146
Urinary potassium (mg/24 h)						
Mid-term vs Baseline	6.25(-39.96 to 52.47)	0.791	-59.26(-106.90 to 11.62)	0.015	-65.52(-131.86 to 0.83)	0.053
Terminal vs Mid-term	-83.66(-135.28 to -32.04)	0.626	-13.03(-65.93 to 39.87)	0.629	70.63(-3.20 to 144.46)	0.061
Terminal vs Baseline	-77.41(-129.09 to -25.72)	0.003	-72.29(-125.27 to -19.32)	0.007	5.11(-68.71 to 78.93)	0.892
